# Unforeseen Effects of Supplementary Feeding: Ungulate Baiting Sites as Hotspots for Ground-Nest Predation

**DOI:** 10.1371/journal.pone.0090740

**Published:** 2014-03-05

**Authors:** Nuria Selva, Teresa Berezowska-Cnota, Isabel Elguero-Claramunt

**Affiliations:** Institute of Nature Conservation, Polish Academy of Sciences, Mickiewicza, Kraków; Université de Sherbrooke, Canada

## Abstract

Despite the ubiquity and magnitude of food provision to wildlife, our understanding of its ecological effects and conservation implications is very limited. Supplementary feeding of ungulates, still one of the main paradigms of game management in Europe, occurs in natural areas on an enormous scale. We investigated the indirect effects of this practice on nest predation risk in the Polish Eastern Carpathians (Bieszczady Mountains). We hypothesized that the predators attracted to ungulate baiting sites would also forage for alternative prey nearby, increasing the nest predation risk for ground-nesting birds in the vicinity. We conducted a paired experiment by placing artificial nests (N = 120) in feeding and control sites (N = 12) at different distances from the ungulate feeding site. We also documented the use of three ungulate feeding sites by potential nest predators with automatic cameras. The proportion of depredated nests was 30% higher in the vicinity of feeding sites than at control sites (65%±31.5 vs 35%±32.1). The probability of a nest being depredated significantly increased with time and at shorter distances from the feeding site. We predicted that the area within 1-km distance from the feeding site would have a high risk (>0.5) of nest predation. We recorded 13 species of potential ground-nest predators at ungulate baiting sites. Most frequent were Eurasian jays *Garrulus glandarius*, mice and voles *Muroidea*, ravens *Corvus corax*, brown bears *Ursus arctos*, and wild boar *Sus scrofa*. Nest predators made most use of supplementary feeding sites (82% pictures with predators vs 8% with ungulates, the target group). Our study alerts of the impacts of ungulate feeding on alternative prey; this is of special concern when affecting protected species. We urge for a sensible management of ungulate feeding, which considers potential indirect effects on other species and the spatial and temporal components of food provision.

## Introduction

Supplementary feeding of wildlife is occurring on a colossal scale nowadays. Food supplementation has been widely used across the globe as a conservation and management tool, particularly for threatened species. Some examples include vulture restaurants, used for more than four decades in Africa, Europe, Asia and North America [1]; winter hay feeding of European bison *Bison bonasus* since the 18^th^ century, and then during the restoration of the species into the wild in the 50s [2]; or feeding programmes of trophic specialists, like the Spanish imperial eagle *Aquila adalberti* [3] or the critically endangered Iberian lynx *Lynx pardinus* [4], after the collapse of their main prey. The provision of food to facilitate wildlife observations as a touristic attraction is also increasing together with a rapidly growing ecotourism industry [5]–[8]. Another recreational purpose is backyard bird feeding, whose popularity has increased over the last decades to the point that there is one feeder every nine birds estimated in the UK [9], and householders purchase half million tonnes of birdseed annually just in the USA and UK [10]. Supplementary feeding and baiting of game wildlife have even a longer tradition and since the last century have been common practices, mainly in Europe and North America [11]–[13]. Only in the Czech Republic, 83,367 ungulate feeding sites were reported in 2004 outside national parks [14].

Despite the ubiquity and magnitude of supplementary feeding practices in wildlife management, our understanding of the ecological effects and conservation implications of these food subsidies is still very limited [10]. As food availability is one of the main factors limiting animal populations [15], artificial feeding has been shown to bring direct benefits, like enhanced survival and reproductive performance [3], [16], [17]. However, these practices can be ineffective [18] or prove detrimental in the long-term [19]–[22]. Supplementary feeding also affects social and territorial behaviour, intra- and interspecific interactions, and animal movements and activity patterns [8], [23], [24]. The indirect effects of supplementary feeding have received comparatively less attention. Feeding sites seem to play a role in disease transmission [10], [25], [26] and the spread of exotics [27], and can initiate trophic cascades. The concentration of scavengers at carrion dumps and vulture restaurants diminishes the presence of alternative prey species of facultative scavengers and increases the probability of nest predation in their surroundings [28], [29]. Backyard bird feeding has also been shown to increase predation on arthropod prey in the area [30], [31]. Herbivore concentrations around feeding sites are associated with overgrazing of the palatable vegetation and changes in the plant composition in the area [11], [19], [27]. These cascading effects may get special relevance when food subsidies are provided in natural and semi-natural areas and when they affect species of conservation concern.

Supplementary feeding of ungulates is still one of the main paradigms of game management in Europe. With the exception of the Netherlands and some Swiss cantons, where it is forbidden, ungulate feeding is practiced in all European countries. It is even obligatory by law and, therefore, conducted intensively, in most central European countries [12]. In North America, this practice has been intensively debated [32], particularly in relation to disease transmission risk [25], [26], and has been widely restricted or prohibited [13]. The goals of this practice have been to maintain high densities of animals for hunting; to improve their nutritional status, survival and reproductive performance, especially in winter, as well as the quality of trophies; to prevent damages in forestry and agriculture; and to attract ungulates to shooting spots or for recreation [11]–[13]. As supplementary feeding, baiting also implies the provision of natural or non-natural food to wildlife. Although the management goals of ungulate supplementary feeding and baiting differ (see definitions in [13], [26]; baiting is rather oriented to hunt or capture the animal), from an ecological perspective, these practices are equal and have similar indirect effects. Therefore, we have treated them indistinctly in this paper.

Here we investigated the effects of ungulate supplementary feeding on the predation risk of ground-nesting birds in the Carpathian Mountains, where this practice is a deeply rooted tradition and obligatory by law [12]. In Poland, it has dramatically increased in the last decades: recent estimates yield about 143 million tonnes of food supplied annually to ungulates in the Polish forests [33]. Ungulate feeding commonly commences in the end of summer and continues till mid spring, though sometimes it extends beyond the period of food shortage. This practice involves simply the establishment of feeding sites or places where the food is regularly thrown on the ground. Selective feeders are not used. These food subsidies may attract target species, some of them trophic generalists, like the wild boar *Sus scrofa*, as well as non-target species, including predators [34], [35]. Therefore, by subsidizing predators and increasing their pressure in the area, ungulate feeding may have an impact on prey species [34], [36]. We hypothesized that the predators attracted to the ungulate bating sites would also forage for alternative prey nearby, increasing the nest predation risk for ground-nesting birds in the vicinity. We documented a significant negative effect of ungulate feeding sites on nest predation risk.

## Materials and Methods

### (a) Ethics statement

The field study did not involve endangered or protected species. No animals were harmed, captured or handled in this study; the methods employed were non-invasive. No samples were collected. The field study was done in strict accordance with legal requirements in Poland. It was conducted in the public forest lands, managed by the Polish State Forest Administration. Research and motorized access of scientists to the public lands managed by the State Forest is guaranteed by the Polish Law on Forest from 28 September 1991. Photo-monitoring of supplementary feeding sites and taking pictures of protected species in public lands with automatic cameras do not require any permit in Poland. In spite of this, agreement from the Forest Districts in the study area was additionally guaranteed previous to the installation of the automatic cameras in the feeding sites.

### (b) Study area

**Figure 1:**
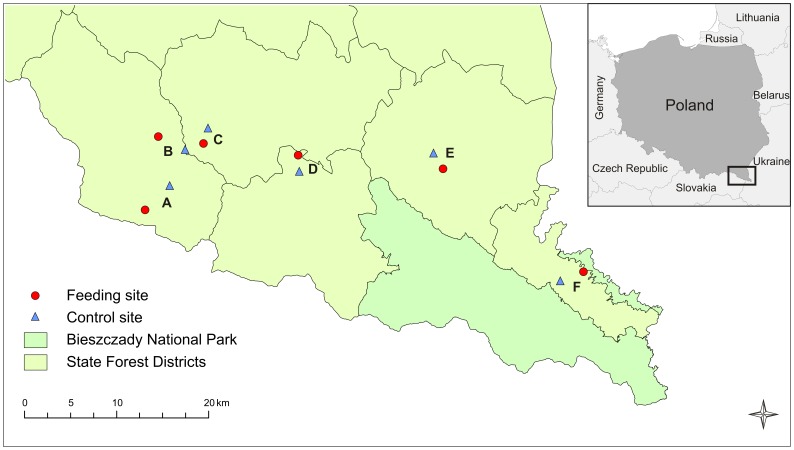
Study area and location of the lines of artificial nests. Map of the Bieszczady Mountains (SE Poland), located in the Northern Carpathians. The nest predation experiment was conducted in the beginning of May 2011 for 15 days in the areas indicated (A–F). In each area, an ungulate feeding site (red circles) and a control site (blue triangles) were selected. State Forest Districts: Komańcza (A, B), Baligród (C), Cisna (D), Lutowiska (E) and Stuposiany (F).

The study was carried out in the Bieszczady Mountains (c.a. 2000 km^2^, SE Poland, Fig. 1), located in the North-Eastern part of the Carpathians. Bieszczady is characterized by mountains of middle and lower altitude (between 400 and 1400 m a.s.l) and gentle slopes [37], [38]. The typical vegetation is the mountain forest, dominated by beech *Fagus sylvatica* and fir *Abies alba*, with admixtures of Norway spruce *Picea abies* and intertwisted with valleys and meadows (between 500 and 1150 m a.s.l.). Higher locations are dominated by these two conifers. In the zone above the upper tree line (called “polonina”, >1150 m a.s.l.), alpine meadows and subalpine grass and shrub communities are typical. The climate is continental. Winters can be quite severe with temperatures dropping below −30°C. Snow cover persists for about three months. The average annual air temperature is 4.9°C [37], [38].

The vertebrate community of the Bieszczady Mountains, with 284 documented species [38], is very rich. It includes five ungulate species (red deer *Cervus elaphus*, roe deer *Capreolus capreolus*, wild boar, European bison, and moose *Alces alces*, this last quite rare), three species of large carnivores (brown bear *Ursus arctos*, Eurasian lynx *Lynx lynx* and wolf *Canis lupus*), and at least 19 species of birds of prey [38]. From the 165 bird species breeding in the study area, tetraonids are represented by the hazel grouse *Tetrastes bonasia*, which is quite common. The black grouse *Tetrao tetrix* and capercaillie *Tetrao urogallus* are present in other areas of the Polish Carpathians and are among the ground-nesting birds of major conservation concern [39], [40]. The Bieszczady Mountains have a significant value for biodiversity conservation. They are part of the Natura 2000 network and the East Carpathians UNESCO MAB Reserve. The Bieszczady Mountains include a protected part (the Bieszczady National Park c.a. 300 km^2^); the rest of the area, exploited by the State Forest Administration, undergoes timber harvest and game management.

The study was conducted in the exploited part of the forest, specifically in the Forest Districts of Baligród, Cisna, Komańcza, Lutowiska and Stuposiany (c.a. 900 km^2^, Fig. 1). In this commercial part, supplementary feeding of ungulates is a game management practice conducted almost year-round, though more intensively in winter. The feeding sites are located inside the forest and consist usually of a small glade or more open forest where supplementary food (maize, beetroots, fodder, grain) is more or less regularly thrown on the ground in the proximity of hunting towers. These sites are typically used for many years. The aim of this practice is to both feed and attract game to these shooting spots [41]. The magnitude of this practice is high, in both the amount of food provided and the density of feeding sites. A total of 170 feeding sites has been inventoried in the study area (Selva et al. unpublished data). The annual amount of supplementary food provided by the State Forest Administration in these five Forest Districts during the hunting seasons of 2010/2011 and 2011/2012 was 614 and 787 tonnes, respectively (data from the Regional Directorate of the State Forest Administration in Krosno).

### (c) Nest predation experiment

We designed a paired experiment with artificial nests in which we distributed them in lines, one close to a feeding site and one at a control site in each area (Fig. 1). We selected six feeding sites located in different areas, where ungulate food was supplied throughout all the year and that were at least 2 km apart from neighboring feeding sites. Within the same area, a control site was chosen for each feeding site within a mean distance of 2.5 km (range: 1.7–3.7), on the basis of habitat similarity (forest type, age and openness, and altitude) and taking into account that no other feeding site was located closer. The proximity of public roads and human settlements were avoided in the selection of sites for the experiment. The lines were located inside the forest, and the nests within each line were distributed at increasing distances from the corresponding feeding site (see distances in Table S1). The distance from each nest in the line to the corresponding feeding site was measured from the central point where the supplementary food was supplied with a handheld GPS. Feeding sites were supplied with maize prior to the start of the experiment and during the first inspection in order to standardize food conditions.

**Figure 2:**
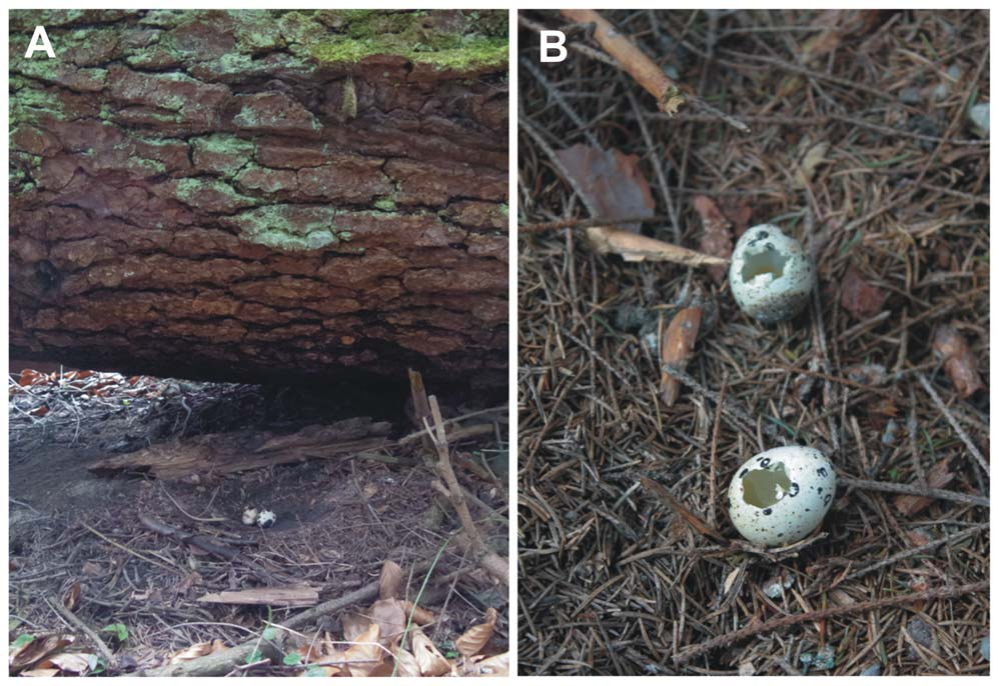
Artificial ground nest. Each nest consisted of two Japanese quail (*Coturnix coturnix*) eggs and imitated the nests of hazel grouse (*Tetrastes bonasia*, A). Nests (N = 120) were checked 6 and 15 days after deployment. A nest was considered depredated if at least one egg was damaged or missing (B). Photos: Nuria Selva, Anne Scharf.

The experiments were conducted during May 2011, coinciding with the bird nesting season. At each site, ten artificial nests, each consisting of two Japanese quail (*Coturnix coturnix*) eggs, were set out in a line, imitating the nests of hazel grouse, a ground-nesting bird species common in the study area [42]. Hazel grouse nests were simulated by scraping vegetation and litter from the soil surface and making a small depression in the ground where two eggs were placed (Fig. 2). The nests were placed at the base of standing large trees or large fallen logs or trees, and at about 50% of tree canopy cover. Eggs were kept refrigerated until the day they were deployed in the field. We used latex gloves and rubber boots during handling to reduce human scent. In any case, the amount of scent tracks that we could leave would be similar in both feeding and control sites. Nests at a given site were placed about 25–30 m away from each other by step counting. GPS coordinates were taken at each nest and the distance to the middle point of the feeding site measured. A spruce branch was placed at the tree or log near each nest to facilitate relocation. To control for potential effects of the features of the nest location, each nest was assigned to one of the two location categories: tree base or fallen log/tree. A total of 120 nests were deployed and checked (Table S1). The artificial nest experiment started on 2^nd^ May in areas A–D and on 3^rd^ May in areas E–F, dates when the eggs were exposed. To minimize disturbance, nests were checked twice: 6 and 15 days after their placement (on 8^th^ and 17^th^ May in areas A–D, and on 9^th^ and18^th^ May in areas E–F). A nest was considered depredated if at least one egg was damaged or missing (Fig. 2). The eggs were not replaced. Signs of potential nest predators were noted whenever observed.

### (d) Identification Of Potential Predators

Half of the feeding sites where the nest experiments were conducted (areas B, C and F; Fig. 1) were continuously monitored with automatic cameras (Reconyx Rapid Fire Professional) to document the presence of potential predators of ground nests. The photo-monitoring of these sites started on 25^th^ April, one week before the nest predation experiment was initiated, and it was conducted until the end of May, which corresponds to the hazel grouse's egg-laying and incubation period in Southern Poland [43]. The cameras were checked (batteries and memory card changed) in the days of nest placement or inspection. In order to obtain a statistically balanced data, the automatic cameras were programmed to take a single picture every 5 minutes. This design was preferred to the triggering program for several reasons: (1) to avoid biases related to species of larger size triggering the camera more often, and then being over-represented, and (2) because feeding sites cover a surface larger than the area of detection of camera motion sensors, therefore animals in the feeding site, but far away from the camera would also be registered. Each photograph taken by the automatic cameras was considered a record. The picture database included date, time, species presence/absence and number of individuals recorded. For each species of potential ground-nest predator, we reported the total number and mean proportion of pictures with the species present (calculated from the total number of positive pictures, i.e. those with species, genus or family identified, and then averaged for the three sites); the mean and maximum number of individuals observed; and, the percentage of camera-trap days when the species was recorded.

### (e) Statistical Analysis

We used generalized linear mixed models (GLMMs) to relate nest predation (1/0, N = 120 nests) to the distance to the feeding site (meters, log-transformed), number of days elapsed since nest exposure (6 and 15 days) and type of simulated nest (at the base of standing tree N = 61, or by a log or fallen tree N = 59). The random term was the line at feeding and control sites (N = 12) nested in area (A–F, N = 6). Models were fitted in R (version 3.0.2) [44] with the lme4 package [45] (function glmer) using a logit link function and a binomial error distribution. Model terms that were not significant were eliminated in a backward stepwise procedure and the final model included only significant effects (p<0.05).

## Results

**Figure 3:**
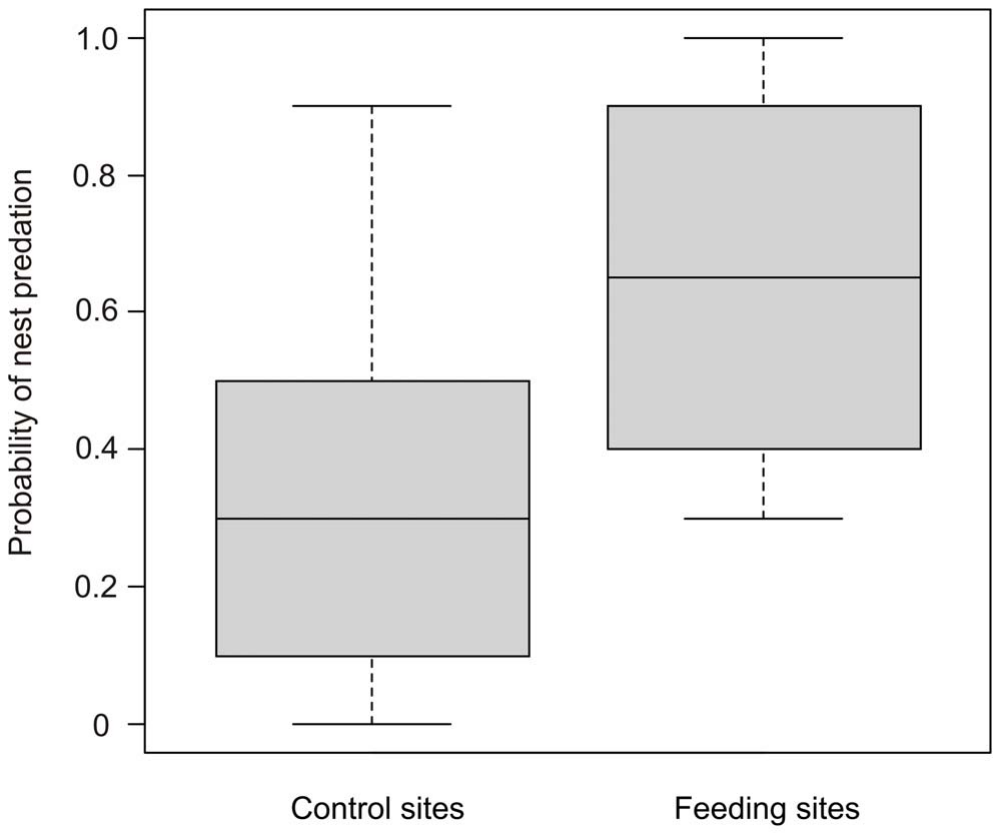
Nest predation at ungulate feeding sites and control sites observed in the field experiment (May 2011). Boxplot of the probability of a nest being depredated in the vicinity of feeding sites (N = 6, between 9 and 108 m) and far away from ungulate feeding sites (N = 6, between 1.7 and 3.7 km). It shows field data as the proportion of depredated nests recorded at the end of the experiment, i.e. 15 days after nest deployment.

**Figure 4:**
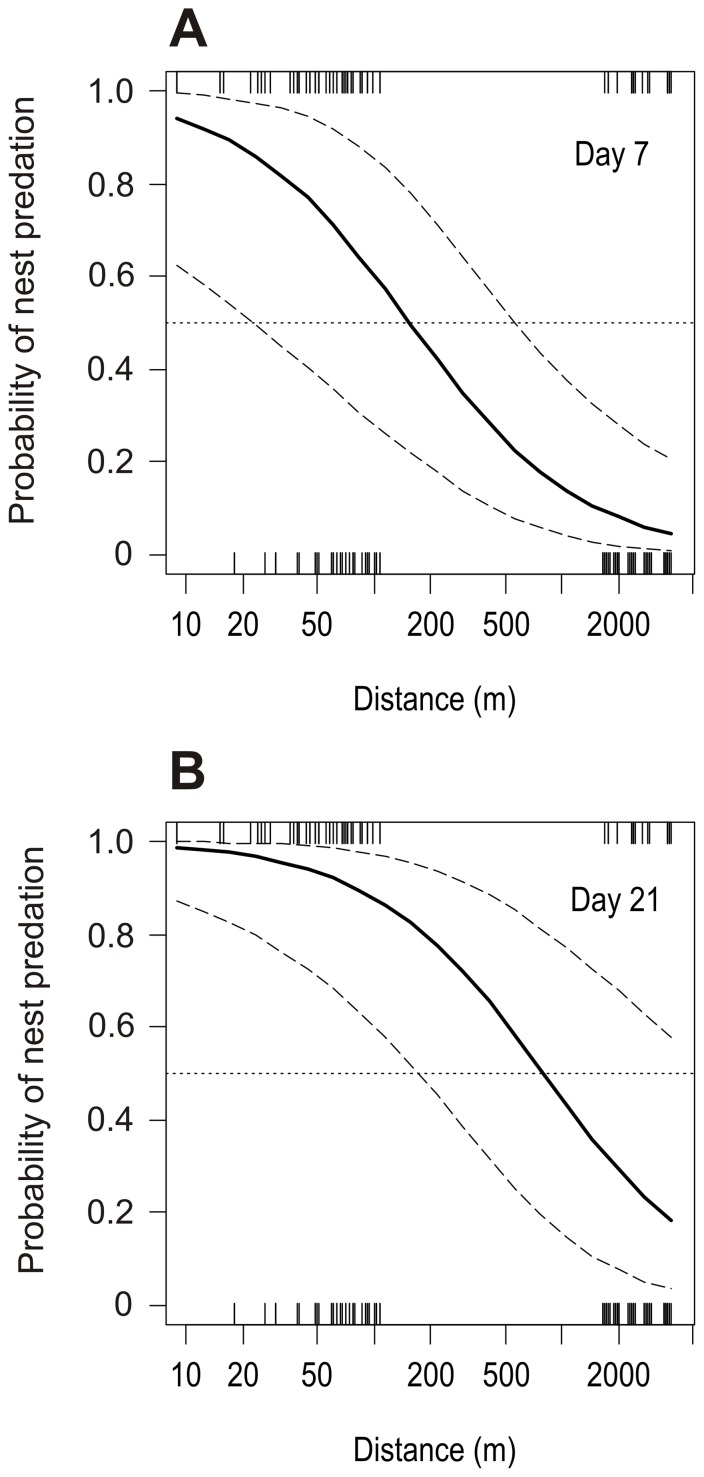
Predicted probability of nest predation in relation to the distance to ungulate feeding sites and time of nest exposure. Estimates of the probability of nest predation with 95% confidence intervals (dashed lines) generated from the logistic model without random effects (Table 1, GLMM fitted to the field data using a logit link function and a binomial error distribution) as a function of the distance to the closest ungulate feeding site and the time elapsed since nest deployment. The effect of the time of nest exposure on nest predation probability is shown for two periods: A) 7 days and B) 21 days. Nest predation risk increased at shorter distances from ungulate feeding sites and with the number of days elapsed since nest deployment. Considering a typical incubation period of three weeks (B), model predictions indicate that the area within 1-km distance from ungulate supplementary feeding sites would have a high probability of nest predation (>0.05). Field data from the experiment is provided as a rug (small ticks inside the box) in the x-axis. Note the logarithmic scale in the x-axis.

The proportion of nests lost to predation was significantly higher (Wilcoxon matched pairs test, W = 35.5, *p* = 0.036) and almost double in the vicinity of ungulate feeding sites than at control sites (Fig. 3). After 6 days, 52% (SD±38.7) of the nests were depredated in the lines close to feeding sites, whereas in control sites only 20% (SD±16.7) of the nests were lost to predators. These proportions increased with time, and after 15 days, 65% (SD±31.5) and 35% (SD±32.1) of the nests were depredated in the feeding and control sites, respectively (Fig. 3). GLMM analysis showed that the distance to the feeding site and the number of days elapsed since nest exposure were the main factors affecting nest predation (Table 1). The probability of a nest being depredated significantly increased with time and at shorter distances from the feeding site. The type of nest and the interaction between the distance to the feeding site and the number of days elapsed since nest deployment did not have a significant effect on nest predation probability. Model predictions suggest that although the effect of feeding sites on the probability of a nest being depredated is a rather local effect (few hundred meters, Fig. 4) when the period of nest exposure is short, it significantly increases with time. Therefore, considering a typical incubation period of three weeks, all area within a 1-km radius from the feeding site would have a high risk of nest predation (>0.05, Fig. 4).

**Table 1:** Factors affecting ground-nest predation.

Fixed effect	Estimate	Lower CI	Upper CI	*p*
Intercept	4.041	0.781	8.071	0.0157
Distance to feeding site	−0.956	−1.636	−0.456	0.0002
Days elapsed since exposure	0.112	0.025	0.207	0.0041

Results of the Generalized Linear Mixed Model explaining the variation in the probability of artificial ground nests (N = 120) being depredated in relation to the distance to ungulate feeding sites (m, log-transformed) and the number of days elapsed since nest deployment (6 and 15 days). The nest line at feeding and control sites (N = 12) nested in the area (N = 6) were included as random factors. Model fitted using a binomial error distribution and a logit link function (package lme4, R version 3.0.2). Only significant effects were retained (*p*<0.05). Estimates of 95% confidence intervals (CI) are based on bootstrap with 1000 simulations.

doi:10.1371/journal.pone.0090740.t001

We processed 26,249 pictures, obtained during 93 camera-trap days at three feeding sites during bird breeding period. We excluded 18 camera-trap days in which no pictures were obtained due to a technical error. We could identify wildlife to species, genus or family (including superfamily) in 5,122 pictures (19.5% of the total). For simplicity, we refer to them as species hereafter. Eighty-two per cent (4,178 pictures) of these positive pictures -where the species was identified- corresponded to potential egg predators. We recorded a total of 13 species of potential ground-nest predators at ungulate baiting sites, including nine mammalian and four avian species (Table 2). All these species are common in the study area [38]. We registered visits of woodpeckers *Picidae*, rodents *Muroidea* and a small mustelid *Mustelidae*; however the quality of images did not allow for proper species identification. The most frequent species of potential nest predator at the supplementary feeding sites was the Eurasian jay *Garrulus glandarius*, detected at 42% of the positive pictures and in 86% of the camera-trap days, followed by mice and voles, present in 68% of the camera-trap days (Table 2). The brown bear, the common raven *Corvus corax*, and the wild boar were also frequent at ungulate feeding sites, and were registered in 11, 8 and 6% of the positive pictures, respectively. Other visitors and potential egg predators included carnivores, such as the Eurasian badger *Meles meles*, the red fox *Vulpes vulpes*, the raccoon dog *Nyctereutes procyonoides*, and the wolf; red squirrels *Sciurus vulgaris*; and, the common buzzard *Buteo buteo*. In general, the most frequent species were also the most gregarious. Particularly the wild boar used to attend the feeding sites in large groups (Table 2). Ungulates, the target group of supplementary feeding practices, were recorded on 287 pictures, representing 7.9% (SD±7.68) of the positive pictures. When excluding the wild boar from the ungulate pictures, large herbivores were present just in 62 pictures (1.52% of the positive pictures, SD±1.834).

**Table 2:** Use of ungulate feeding sites by potential nest predators during bird nesting season.

Species	No. feeding sites	No. pictures	Photographic records (mean ±SD)	Camera-trap days (%)	Mean no. individuals (max)
Eurasian jay *Garrulus glandarius*	3	2315	42.21±12.872	86.0	1.4 (7)
Mice and voles *Muroidea*	3	534	12.0±14.447	67.7	1.4 (7)
Common raven *Corvus corax*	2	505	7.92±7.018	47.3	1.8 (5)
Brown bear *Ursus arctos*	3	479	10.74±10.722	40.9	1.1 (2)
Wild boar *Sus scrofa*	3	225	6.40±6.029	24.7	2.0 (11)
Eurasian badger *Meles meles*	2	164	2.05±2.800	21.5	1.0 (2)
Woodpeckers *Picidae*	1	51	0.58±1.002	21.5	1.1 (2)
Red squirrel *Sciurus vulgaris*	2	20	0.61±0.893	16.1	1.0 (1)
Red fox *Vulpes vulpes*	2	8	0.11±0.120	6.5	1.0 (1)
Raccoon dog *Nyctereutes procyonoides*	1	3	0.09±0.157	2.2	1.0 (1)
Grey wolf *Canis lupus*	1	3	0.09±0.161	2.2	1.0 (1)
Common buzzard *Buteo buteo*	1	3	0.03±0.059	3.2	1.0 (1)
Unidentified small mustelid *Mustelidae*	1	1	0.03±0.054	1.1	1.0 (1)

Results from photo-monitoring of three feeding sites during 93 camera-trap days (25 April- 31 May 2011) showing the number of feeding sites visited by the species, the total number and the mean proportion of pictures with the species recorded (from the total of positive pictures, i.e. with species identified), the proportion of camera-traps days in which the species was photographed, and the mean and maximum (in brackets) number of individuals of a given species registered.

doi:10.1371/journal.pone.0090740.t002

## Discussion

We provided experimental evidence of an increase in the predation risk of artificial ground nests close to ungulate feeding sites. By attracting and concentrating nest predators in their vicinity, supplementary feeding sites can become predation hotspots. This is in agreement with other studies showing that the concentration of food subsidies redistributes local predators [28],[46],[47]. By aggregating predators, these subsidies increase the top-down effect of predation on alternative prey [28],[29],[31]. The magnitude and spatial extent of this effect may be stronger when the supplementary food is provided for long periods. This may be related to the fact that the probability of alternative prey being depredated increased with time ([31], this study). Experimental food additions for one month significantly increased predation risk in nests <50 m from feeders and also predator abundance in areas <100 m from feeders [47]. In the case of long-lasting ungulate carcasses, hares *Lepus europaeus* had a higher risk of encounter foxes within 1-km distance from those carcasses [28]. In our area, ungulate feeding sites have been used for decades and are supplied with food almost year-round. Our model indicated that the areas within 1-km distance from feeding sites had a probability of nest predation higher than 0.5 during the incubation period. Considering the large number of ungulate feeding sites in the study area, about one fifth of the area is estimated to have a high nest predation risk.

Our findings complement those of Cooper and Ginnett [34], who also found a lower survivorship of artificial nests close to deer feeders in North America. That study was conducted in a different habitat, mainly pastures for cattle grazing and in sites with open water, and simulated larger ground-nesting birds. Interestingly, the increase in nest predation rates obtained at feeding sites in relation to control sites was similar in both studies (27.5% vs 30% in our study). Hamilton et al. [36] also found that the survival of artificial turtle nests was 5.5 times higher at lakes without deer feeders. The consistency between these studies and our results indicates that the indirect negative effects of ungulate supplementary feeding practices on alternative prey, and particularly on ground-nesting species, may be more widespread than previously thought.

Artificial nests have been widely used in ecological studies, and in spite of their limitations in reflecting natural patterns [48], they often represent the only tractable way to get a sufficient sample to test hypothesis. They are particularly useful for comparisons among treatments or gradients of environmental conditions, and to investigate predation patterns of rare or endangered species [49]–[51]. In our study, the aim was to assess the effect of feeding practices on nest predation rates while controlling for field conditions as much as possible. Therefore, any potential bias would affect equally control and treatment sites. Moreover, a recent study using natural nests yielded the same results and confirmed the negative effect of food supplementation on nest predation rates [47].

Ungulate supplementary food attracted mostly non-target species. The species making most use of artificial food were potential nest predators (82% vs 8% of the pictures with ungulates). An intensive use of deer supplementary food by non-target species has also been documented in North America, mainly by raccoons *Procyon lotor* and passerine birds [34],[35]. Among the recorded visitors of ungulate feeding sites, squirrels, small mammals (shrews, voles and mice), corvids, mustelids, and canids have been often reported as predators of bird nests [52],[53]. By contrast, ungulates, the target group, were rare visitors, with the exception of the wild boar, which can become an important egg predator [54]. For instance, in some areas the proportion of capercaillie nests lost to wild boar can reach up to 30% [55]. In other areas of the Carpathian Mountains, both wild boar and brown bear have been recorded as frequent predators of hazel grouse and capercaillie nests [55]. Ungulate feeding practices are widely non-selective and may enhance population growth and range expansion of native species into new areas and sensitive habitats, such as the wild boar into alpine environments [56]. They also may promote invasive species which are potential nest predators, such as raccoons and raccoon dogs in Europe [57].

Wildlife management practices that increase nest mortality may come into conflict with the conservation of birds. Predation is one of the main causes of avian nest mortality [52],[58] and its increases can have important consequences for population dynamics [59]. The percentage of ground nests lost to predators has been estimated to average 30.6% in forest habitats and 48.8% in shrubs and grasslands [58]. In forest habitats, this practice may widely affect tetraonid birds, whose populations are seriously declining worldwide or are threatened at local, regional or national scales [40]. For instance, in some areas of the Carpathian Mountains about 65% of the capercaillie and hazel grouse clutches were lost to predators [55]. In open habitats, such as grasslands and wetlands, the impact of ungulate feeding sites may be even stronger, due to the generally higher predation rates in these habitats [58], and the higher probability of nest trampling [60]. Nest predation in European meadow birds has increased by more than 40% in the last decades, and is regarded as one of main reasons for the population decline of these birds [59]. These findings, together with the general increase of ungulate numbers and overabundance of them in many regions, including Poland [12],[41], stress the need to rethink ungulate supplementary feeding practices, particularly in central Europe.

We suggest that avoiding ungulate supplementary feeding sites in breeding areas of endangered species of ground-nesting birds may reduce the risk of nest predation, and should be seriously considered. In addition, removing the food from feeding sites before the start of the bird breeding season, when predation is higher [47],[55], seems also a sensitive and prudent strategy. Both recommendations are in line with those proposed by Cooper and Ginnett [34]. The location of ungulate feeding sites must be carefully selected, taking also into account the potential indirect effects on other species. We encourage further research on the effectiveness of these recommendations, and on the impacts of ungulate feeding sites on the reproductive success of specific bird species undergoing strong population declines. Not only game management, but also nature conservation projects should take into account the indirect effects of feeding practices. Supplementary feeding programs are also widely used in conservation (c.a. 50 European LIFE funded projects, LIFE project database http://ec.europa.eu/environment/life/project/Projects/). On the other side, conservation projects (e.g. for capercaillie conservation) aimed at improving the conservation status of ground-nesting birds often involve the direct reduction of nest predators, while ignoring indirect feeding of these predators through ungulate feeding practices.

Whenever strategies regarding supplementary feeding are to be adopted, the supply of food, and its temporal and spatial availability must be critically examined. The concentration of food supplies at feeding stations is an ecological perturbation, with important consequences for the plant and animal communities in the surrounding areas. This fact must be kept in mind when establishing and managing feeding sites, especially when protected and/or game species may be potentially affected [61]. Our findings remark that wildlife management should consider complex interactions, indirect effects, and community processes. Instead of management being focused on single species or group of species, an ecosystem approach should be favoured.

## Supporting Information

Table S1 **Data from the nest predation experiment conducted in May 2011.** The area (A–F), nest line (a–l), type of site (feeding vs control), location of the nest (at the base of a large standing tree or under a fallen log or tree), air distance to the corresponding feeding site (m), number of days elapsed since the nest was deployed, and whether the nest was predated or not (1/0) in the field inspections. Field inspections were conducted 6 and 15 days after nest deployment. (XLSX)
